# Molecular subtypes, clinical significance, and tumor immune landscape of angiogenesis-related genes in ovarian cancer

**DOI:** 10.3389/fonc.2022.995929

**Published:** 2022-08-29

**Authors:** Haixia Tang, Jingsong Shan, Juan Liu, Xuehai Wang, Fengxu Wang, Suping Han, Xinyuan Zhao, Jinxiu Wang

**Affiliations:** ^1^ Department of Gynecology, Nantong Hospital of Traditional Chinese Medicine, Nantong, China; ^2^ Division of Natural and Applied Sciences, Duke Kunshan University, Kunshan, China; ^3^ Department of Obstetrics and Gynecology, Women’s Hospital of Nanjing Medical University, Nanjing Maternity and Child Health Care Hospital, Nanjing, China; ^4^ Department of Occupational Medicine and Environmental Toxicology, Nantong Key Laboratory of Environmental Toxicology, School of Public Health, Nantong University, Nantong, China; ^5^ Department of Gynecology, The First Affiliated Hospital of Nanjing Medical University, Nanjing, China

**Keywords:** angiogenesis, ovarian cancer, tumor microenvironment, prognostic signature, drug sensitivity

## Abstract

Angiogenesis is a physiological process, where new blood vessels are formed from pre-existing vessels through the mechanism called sprouting. It plays a significant role in supporting tumor growth and is expected to provide novel therapeutic ideas for treating tumors that are resistant to conventional therapies. We investigated the expression pattern of angiogenesis-related genes (ARGs) in ovarian cancer (OV) from public databases, in which the patients could be classified into two differential ARG clusters. It was observed that patients in ARGcluster B would have a better prognosis but lower immune cell infiltration levels in the tumor microenvironment. Then ARG score was computed based on differentially expressed genes *via* cox analysis, which exhibited a strong correlation to copy number variation, immunophenoscore, tumor mutation load, and chemosensitivity. In addition, according to the median risk score, patients were separated into two risk subgroups, of which the low-risk group had a better prognosis, increased immunogenicity, and stronger immunotherapy efficacy. Furthermore, we constructed a prognostic nomogram and demonstrated its predictive value. These findings help us better understand the role of ARGs in OV and offer new perspectives for clinical prognosis and personalized treatment.

## Introduction

Ovarian cancer (OV) is the most common gynecological cancer with the highest mortality rate in the world, accounting for 4.4% of female cancer-related mortality in 2018 (1, 2). Annually, 225,500 new incidences of ovarian cancer are diagnosed worldwide, resulting in 140,200 cancer-specific deaths (2, 3). Due to the heterogeneity of OV, the World Health Organization (WHO) classifies it into several morphological categories based on cell type (4). Despite significant differences in molecular biology and prognosis, they are all treated identically with cytoreductive surgery and platinum/taxane combined chemotherapy (5). Most patients respond favorably to first-line treatment, but most patients relapse and develop chemotherapeutic resistance (6–9). Worse more, OV is insidious with few sentinel symptoms and lack of effective diagnostic strategies (10–12). As result, more than two-thirds patients were diagnosed with a bad prognosis in an advanced stage (13, 14). Many studies have shown that early diagnosis and appropriate treatment can significantly reduce the metastasis and recurrence of OV (11, 15).

Late diagnosis and heterogeneous treatment result in poor clinical outcomes of patients with OV (16). Thus, novel methods of diagnosis and treatment are required. Immunotherapy has been a research hotspot and an essential supplementary cancer treatment method in recent years due to the in-depth understanding of immune recognition and immunomodulatory molecules (17). Molecular subtyping analysis of OV with complex heterogeneity has a promising future due to the development of molecular tools. Researchers around the world are attempting to identify novel biomarkers that combine molecular characteristics with traditional clinicopathological parameters to improve risk stratification systems to predict clinical outcomes and response to immunotherapy (18).

Angiogenesis, one of the hallmarks of cancer, is the formation of new blood vessels from pre-existing ones through a process called sprouting (19). Angiogenesis plays a significant role in supporting tumor growth and progression, where numerous angiogenic factors are often overexpressed (20, 21). Suppression of angiogenesis has been recognized as a promising therapeutic strategy, especially for cancers that are resistant to conventional treatment (22). It is believed that anti-angiogenic therapy could correct anatomical and functional abnormalities in tumor blood vessels through the process called **“**vascular normalization**”** (23, 24). Moreover, this may help prevent cancer cells from developing aggressive phenotypes related to hypoxic microenvironments (20, 25). These studies suggest that exploring the molecular characteristics of angiogenesis-related genes (ARGs) can help clarify the causes of OV heterogeneity and provide new prognostic and therapeutic approaches.

Wang, G., et al. analyzed the molecular subtypes of ARG in Glioblastoma multiforme and established a prognostic model to predict the treatment response of patients  (26). Based on the 48 ARGs provided in their study, we developed a prognosis prediction model in OV, which reveals the significant value in prognosis, tumor microenvironment, and pharmacological sensitivity. Furthermore, we incorporated the ARG score with the clinical characteristics for clinical outcomes prediction and verified its accurate prediction performance. Our research will provide new concepts for accurate diagnosis and personalized treatment of OV patients.

## Methods and materials

### OV dataset and reprocessing

Gene expression data and relevant clinical information of OV patients are obtained from the public databases The Cancer Genome Atlas (TCGA) and Gene Expression Omnibus (GEO). In this study, two cohorts, GSE9891 and TCGA-OV, were used for subsequent analysis, where cases without complete clinical data will be excluded to minimize statistical bias. The details of the sample are displayed in **Table S1**. For differential analysis, FPKM (fragments per kilobase) values of the TCGA-OV cohort were converted to transcripts per kilobase million (27). We combined the TCGA-OV and GSE9891 and corrected the batch effects using the “ComBat” algorithm from the “sva” package (28).

### Consensus clustering analysis for ARGs

Forty-eight ARGs were derived from the previous study (26). According to these gene expression profiles, “ConsumusClusterPlus” was constructed for consumes clustering (29), where patients were divided into various molecular subgroups on the basis of gene expression pattern. For the major parameters in the “ConsensusClusterPlus” function, we set the max clusters number (maxK)=9, repeated times (reps)=1000, proportion of items to sample (pItem)=0.8, proportion of features to sample (pFeature)=1, cluster algorithm (clusterAlg)=hc/hierarchical, distance= spearman (29). Subsequently, the principal component analysis (PCA) was performed by the “ggplot2” R package.

### Identification of Gene subtypes based on DEGs

Firstly, the R package “limma” was used to investigate the differentially expressed genes (DEGs) between distinct clusters with the standard of adjusted p-value < 0.05 (30). Following that, two different gene subtypes were identified with the consistent clustering algorithm. Gene Ontology (GO) and Kyoto Encyclopedia of Genes and Genomes (KEGG) analyses were performed to further investigate the enriched molecular pathway (31, 32).

### Build prognostic risk signature related to angiogenesis

After data reprocessing, OS-related prognostic OV samples were screened out for further analysis. The TCGA-OV cohort served as the training set, while samples from GSE9891 and the set consisting of the TCGA-OV cohort and GSE9891 served as the testing set to validate the performance of the signature. In the training set, correlations between DEGs and OV survival were determined by univariate Cox regression analysis. The R package “glmnet” was then used to perform the least absolute shrinkage and selection operator (LASSO) regression based on *angiogenesis*-related prognostic genes to minimize the risk of overfitting (33). Formula: $risk\;factor=\sum_{i=1}^{n}coef_{i}\times exp_{i}$ was employed to select candidate genes to build prognostic signature based on ARGs using multivariate Cox analysis. The coef and exp respectively refer to the risk coefficient and gene expression level. The patients were classified into high-risk and low-risk groups according to the median risk score. Subsequently, we used the “survminer” software to conduct the Kaplan–Meier analysis of survival. Receiver operating characteristic (ROC) curves were then used to evaluate the model’s precision. The performance of the model precision was then assessed by plotted ROC curves.

### Compared the risk score of different clinical features and stratified analysis

The correlations between risk score and various clinicopathological characteristics (grade, stage, age, fustat, and histological type) were evaluated using univariate and multivariate cox regression analysis, where **Table S2** provides clinical details. We conducted univariate and multivariate cox analyses to investigate whether the risk score is a factor independent of other available clinicopathological features. Furthermore, stratified analysis was also conducted to examine the performance of the model based on the clinical characteristics described before.

### Assessment of immune infiltration level

Cancers relied on their complex tissue environments for sustained growth, invasion, and metastasis. Moreover, drug resistance and tumor recurrence are intimately associated with the tumor microenvironment (TME) as a potential therapeutic target (34, 35). Gene sets of relevant biological processes were curated from previous research (36, 37). From the gene expression pattern of these related pathways, the ESTIMATE algorithm conducted through the R package “estimate” can predict the status of TME (38, 39). Differences in immune function between different subgroups were then demonstrated by single-sample gene set enrichment analysis (ssGSEA), which allows the quantitative evaluation of immune cell components derived from complex gene expression data (40, 41). Subsequently, the abundance of 22 tumor immune infiltrating cells (TIIC) in risk groups was quantified by CIBERSORT.

### Prediction of immunotherapy response

Immunophenoscore (IPS) was utilized to investigate the immunotherapeutic function of immune cell infiltration scores, which has been validated as a predictor of patient immunotherapy response (42). Higher IPS refers to higher immunogenicity. Tumor mutation burden (TMB) represents the number of mutations per megabase of DNA sequence in a given tumor, which can be used to identify patients who will obtain the greatest benefit from immune checkpoint inhibitors (ICIs) (43, 44). The burden of copy number variation (CNV) gain or loss was evaluated by gene pattern (45).

### Drug sensitivity analysis

The half-maximum inhibitory concentration (IC50) was employed to assess the efficacy of chemotherapeutic drugs in OV patients. The CellMiner database served as the drug sensitivity data source, which was created in response to the list of 60 types of cancer cells (NCI-60) compiled by the National Cancer Institute’s Center for Cancer Research (46).

### Construction of a nomograph system

To predict the prognosis of OV based on clinical characteristics and risk score, a nomograph system was constructed to measure the OS of 1-, 3- and 5- years through R package “rms” (47). In the nomogram, each variable is assigned a score, and the total score is obtained by adding the scores of all factors to make an accurate prediction. Next, we conducted the area under the curve (AUC) and c-index to evaluate the prediction capacity of the nomogram (48–50).

### Statistical analysis

All statistical analyses were conducted in R version 4.1.0 with P < 0.05 defined as significant. The difference between the subgroups was determined by the student t-tests and variance analysis. Spearman and distance correlation analyses were used to compute the correlation coefficients between the expression of ARGs and immune infiltrating cells.

## Results

### Genetic mutation landscape of ARGs in OV

**Figure 1:**
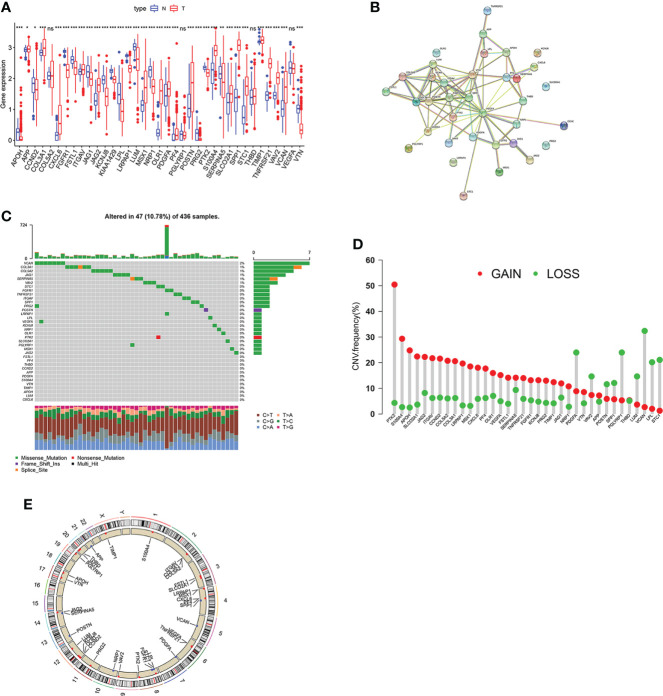
Genetic mutation landscape of ARGs in OV.**(A)** Expression pattern of ARGs in OV and normal tissues. **(B)** The interactivity of DEGs is revealed by PPI analysis. **(C)** genetic alternation of ARGs where mutations occurred in 47 of 436 OV patients. **(D)** CNV gain, loss, and non-CNV frequency in ARGs. **(E)** The chromosomal distributions of CNV alterations in ARGs. Adjusted p-values were shown as ns, not significant; *p<0.05; **p<0.01; ***p<0.001.

The analysis process of this study is shown in **Figure S1**. Firstly, we investigated the different expression pattern of the 48 ARGs in tumor and normal samples within the TCGA-OV dataset (**Figure 1A**). The string website was then employed to conduct a protein-protein interaction (PPI) analysis of DEGs (**Figure 1B**). Subsequently, the incidence of CNVs and somatic mutations of ARGs were analyzed in OV, where 47 mutations occurred in 436 samples with 10.78% somatic mutation. It is observed that VCAN has the highest mutation frequency (2%), followed by COL3A1, COL5A2, and other genes (**Figure 1C**). Furthermore, we investigated the CNV mutational incident, which had significantly increased in genes like PTK2, S100A4, and APOH but decreased in genes like VCAN, PDGFA, and PGLYRP1 (**Figure 1D**). **Figure 1E** displays 48 ARGs’ chromosomal locations of the CNV alterations. Among the 48 genes, 27 ARGs presenting significant prognostic values were identified **(** **Figure S2** **)**. The above results suggest the potential regulatory role of CNV in ARGs expression, which plays an important role in the development of OV.

### Identify ARGclusters in OV

**Figure 2:**
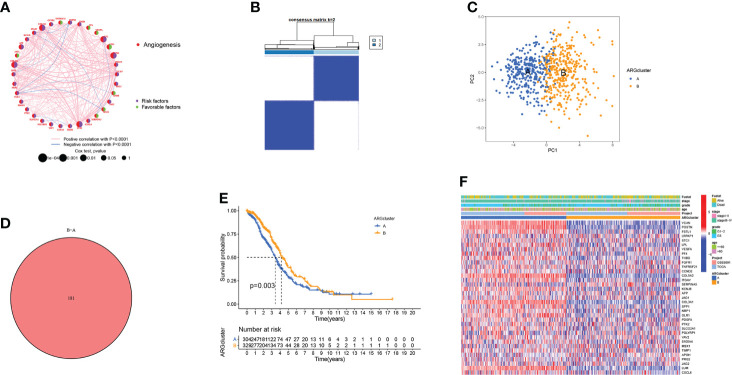
Generation of the ARGclusters in OV. **(A)** The network of interactions between ARGs in the TCGA-OV cohort, where the line thickness indicates the correlation strength. **(B)** The consensus clustering analysis classified samples into two subgroups when k = 2. **(C)** PCA analysis revealed the obvious distinctions in transcriptomes of two subtypes. **(D)** Venn diagram showing the similar parts between two clusters. **(E)** The difference in survival probability between two ARGclusters. **(F)** The distinctions in gene expression levels and clinicopathological characteristics.

In the angiogenesis network, the ARGs interactions, regulator relationships, and their prognostic significance in OV patients were illustrated (**Figure 2A**). To further analyze the expression features of ARGs in OV, we conducted the consensus clustering analysis, where the patients were classified from k = 2 to k = 9 (**Figure S3**). The results revealed that k=2 was the optimal clustering variable (**Figure 2B**). Moreover, PCA analysis also verified the discrepancies between these two ARGclusters (**Figure 2C**). Between the two ARGclusters, there were 181 differently expressed genes (**Figure 2D**). Furthermore, a substantial OS time disparity was detected between the two ARGclusters., where patients in ARGcluster B have a higher survival probability (p=0.003, **Figure 2E**). Then we examined the ARGs expression levels and clinicopathological characteristics between the ARGclusters and identified the distinctions (**Figure 2F**).

### Characteristics of TME in different subtypes

**Figure 3:**
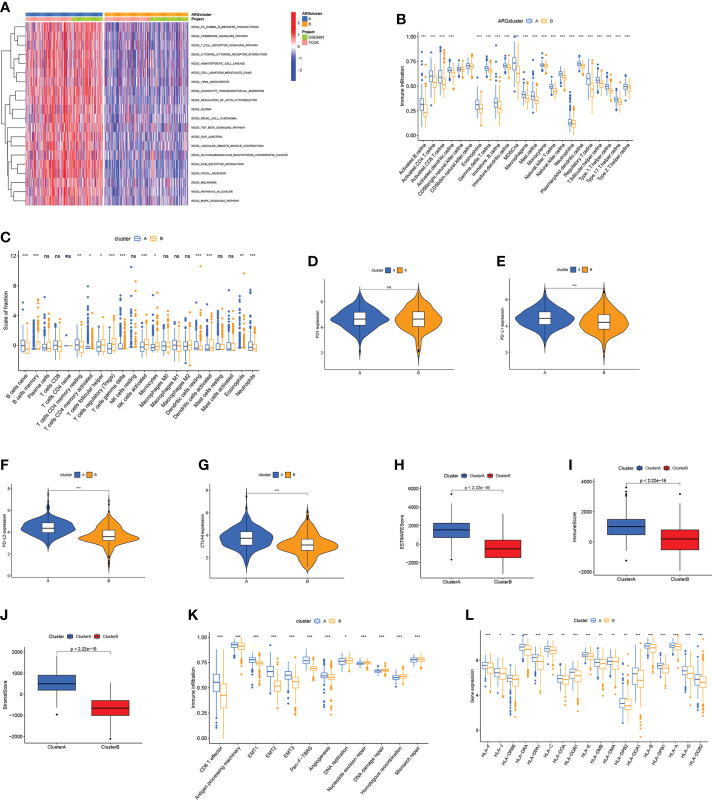
Correlations between TME and ARGclusters. **(A)** GSVA of biological pathways between ARGclusters, where red indicates activation while blue indicates inhibition. **(B)** The abundance of infiltrating immune cells in two ARGclusters. **(C)** 22 TIICs were evaluated by the CIBERSORT algorithm in two ARGclusters. **(D–G)** The expression level of immune checkpoints in two ARGclusters. **(H–J)** Comparison of TME scores in two ARGclusters. **(K)** Correlations between known relevant biological processes and two ARGclusters. **(L)** HLA expression levels in two ARGclusters. Adjusted p-values were shown as ns, not significant; *p<0.05; **p<0.01; ***p<0.001.

According to the gene set variation analysis between these two ARGclusters, it was observed that cluster A was enriched in cancer-related pathways (like Glioma, Renal cell carcinoma, and Melanoma) and metastasis-related pathways (like focal adhesion, cell adhesion molecule, and ECM receptor interaction) (**Figure 3A**). Then ssGSEA was employed to explore the immune infiltration levels in these two ARGclusters, where significant enrichment difference was noticed. The enrichment levels of innate and adaptive immune cells were all significantly higher in ARGcluster A (**Figure 3B**). Subsequently, the correlation between two ARGclusters and 22 TIICs was determined using CIBERSORT **(** **Figure 3C**). We noted that the expression levels of immune checkpoints, PD1, PD-L1, PD-L2, and CTLA4, were all higher in ARGcluster A **(** **Figures 3D–G**). Moreover, it was observed that ARGcluster A has higher TME scores **(** **Figures 3H–J**). ARGcluster A is usually identified as “hot” tumors characterized by strong immune infiltration levels that will benefit more from the immunotherapy, while ARGcluster B can be characterized as a “cold” tumor with low intensity of immune infiltration and relatively unsuitable for immunotherapy. Furthermore, we explore the correlation between known biological processes and these two ARGclusters, where some immune-related processes like CD8 T effector, antigen processing machinery, and Pan−F−TBRS were prominent in ARGcluster A (**Figure 3K**). Additionally, ARGclusters A also had markedly higher expression levels of human leukocyte antigen (HLA) related genes (**Figure 3L**).

### Identification of gene subtype based on DEGs

**Figure 4:**
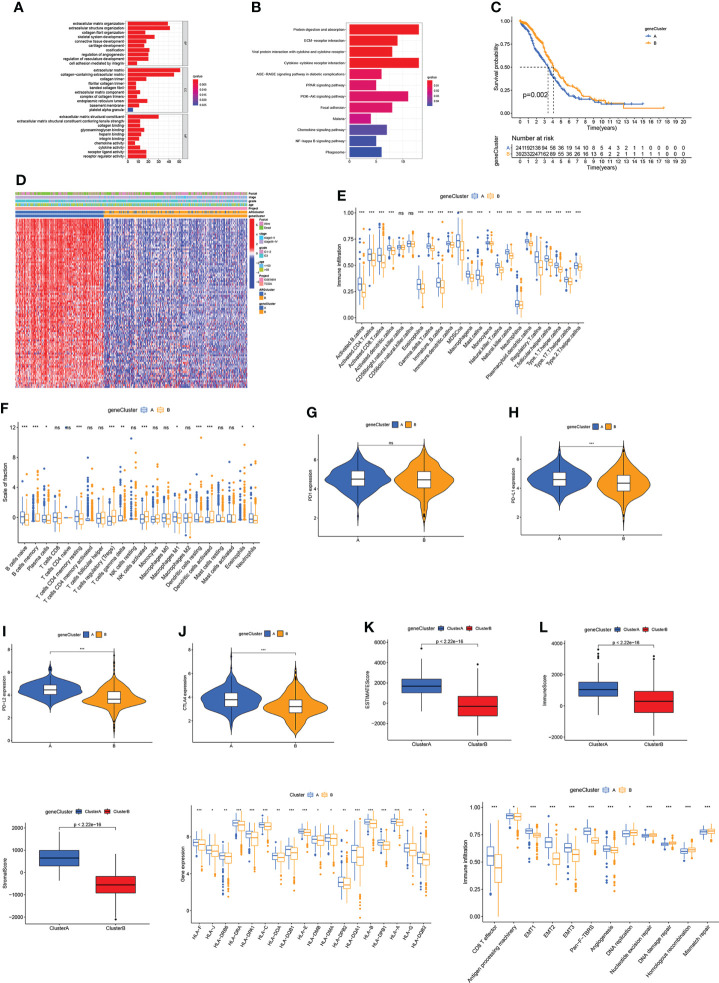
Identification of gene subtype based on DEGs. **(A, B)** GO and KEGG enrichment analysis. **(C)** Kaplan-Meier curve for OS of OV patients. **(D)** Correlation between two gene clusters and clinicopathologic features. **(E, F)** Immune infiltration levels in two gene clusters. **(G–J)** Immune checkpoints expression levels in two gene clusters. **(K–M)** TME scores in two gene clusters. **(N)** HLA expression levels. **(O)** The scores of immune infiltrations. Adjusted p-values were shown as ns, not significant; *p<0.05; **p<0.01; ***p<0.001.

The “limma” package was employed to conduct functional enrichment analysis and screen out the DEGs between two ARGclusters. GO and KEGG analysis revealed that DEGs between two ARGclusters were primarily enriched in immune-related pathways, indicating their importance in the immunological regulation of TME. (**Figures 4A, B** **)**. Subsequently, univariate COX analysis and consensus clustering analysis were employed to categorize the samples into different clusters based on the DEGs in OV patients. The results indicated that the clustering effect was the best when k=2 (**Figure S4**). Kaplan-Meier curve demonstrated that patients in gene cluster B had a higher survival probability (*P* = 0.002; **Figure 4C**). Patients in ARGcluster B are basically patients of gene cluster B, which was associated with better survival status, and early-stage (**Figure 4D**). The immune infiltration levels in these two gene clusters were investigated by ssGSEA, where gene cluster A has a higher enrichment level of immune cells (**Figure 4E**). Additionally, the results of CIBERSORT algorithm revealed that gene cluster A was primarily infiltrated by adaptive immune cells like B cells naive and macrophages M1 (**Figure 4F**). Moreover, the expression of immune checkpoints and TME scores were all higher in gene cluster A (**Figures 4G–M**). Gene cluster A also has higher expression levels of HLA related genes and classical biological pathways like CD8 T effector, EMT and Pan-F-TBRS were more prevalent (**Figures 4N, O** **)**. The above immune signatures indicate that gene cluster A can be defined as a “hot” tumor.

### Establish and validate the prognostic model based on ARG score

**Figure 5:**
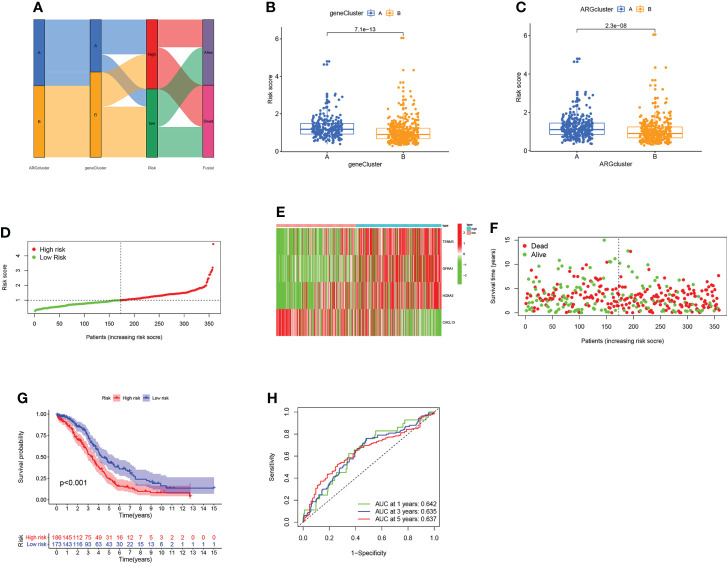
Construction of the ARG score in the training set. **(A)** The dispersion of patients. **(B)** Difference of the risk score in two gene clusters. **(C)** Difference of the risk score in two ARGclusters. **(D)** Distribution of risk scores in two groups. **(E)** Expression pattern of four ARGs in two groups. **(F)** The fustat of patients. **(G)** The comparison of the OS between two groups. **(H)** The sensitivity and specificity of 1-, 3- and 5-year survival rates were predicted based on ARG scores.

To estimate the prognosis of individual OV patients, we developed an ARG scoring system based on these DEGs. **Figure 5A** shows the distributions of the patient in two ARGclusters, two gene clusters, and two risk score groups. To establish the optimal predictive model, LASSO and multivariate cox analysis were performed on the DEGs of 317 samples from the training set (**Figure S5**). We finally screened out four genes (TENM3, GFRA1, HOXA3, and CXCL13) associated with the OV survival were screened out based on the minimum partial likelihood deviation and multivariate cox regression analysis. The ARG score can be calculated as following: Risk score = (0.127* expression of TENM3) + (0.1368* expression of GFRA1) + (0.1358* expression of HOXA3) + (-0.1879* expression of CXCL13). It is observed that the risk score of gene cluster B and ARGcluster B was significantly lower (**Figures 5B, C** **)**. The patients were divided into a high-risk group and a low-risk group based on the median risk score (**Figure 5D**). Moreover, the expression patterns of four genes in the two groups were shown in the heatmap, and the fustat of patients was shown in the scatter plot (**Figures 5E, F** **)**. Patients with low-risk scores had better OS performance than those with higher scores (P < 0.001; **Figure 5G**). The AUC values of ROC curves for 1-, 3-, and 5-year survival rates were 0.642, 0.635, and 0.637, respectively (**Figure 5H**). Then the above results were validated by using the GEO cohort and data set comprised of the GEO cohort and TCGA cohort as the testing set (**Figure S6**).

### Compared the risk score of different clinical characteristics and stratified analysis

The risk scores of individuals with various clinicopathological characteristics were examined to determine their association. It was observed that a higher risk score corresponds to worse fustat status and advanced stage (**Figures S7A–D**). Subsequently, Cox regression analysis of risk score and clinical characteristics (age, grade, and stage) illustrated that risk score was an independent prognostic factor for OV patients (**Table S2**). Following that, we conducted the subgroup analysis to validate the prediction capacity of the signature. As depicted in **Figures S7E–J**, except for the patients with stage I- II, the survival outcomes of the high-risk score group were worse than that of the low-risk group, regardless of their clinical features.

### Estimation of TME based on ARGs

**Figure 6:**
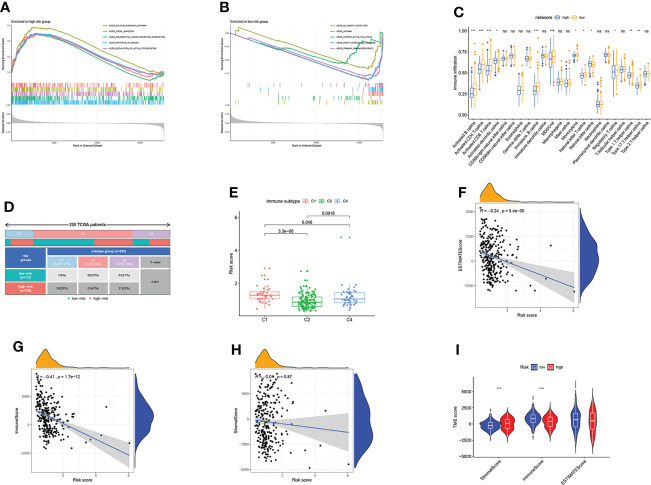
*Estimation of TME based on ARGs.* **(A)** GSEA of high-risk score group. **(B)** GSEA of low-risk score group. **(C)** The difference in immune infiltration levels. **(D)** 220 TCGA patients were divided into three immune subgroups. **(E)** Risk scores of three immune subgroups. **(F–H)** Correlations between risk scores and estimated scores, immune cells, and stromal cells. **(I)** Comparison of TME scores in two ARG score groups. Adjusted p-values were shown as ns, not significant; *p<0.05; **p<0.01; ***p<0.001.

**Figure 7:**
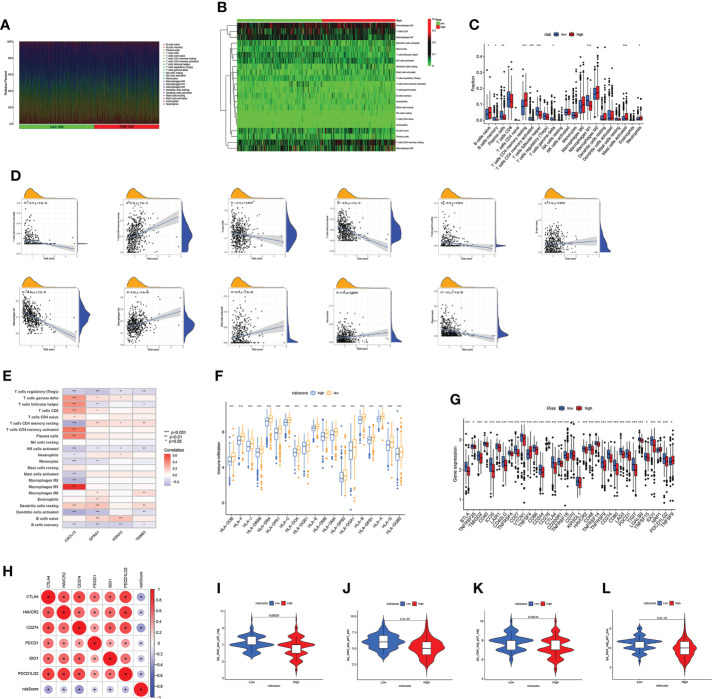
*Immune infiltration characteristics of the two subgroups.* **(A–C)** Differences in immune cell abundance. **(D)** Correlations between risk scores and immune cell abundance. **(E)** Correlations between selected genes in prognostic model and immune cell abundance. **(F)** The expression level of HLA in two risk groups. **(G)** Differential expression of thirty-four immune checkpoints in the two subgroups. **(H)** Correlations between selected immune checkpoints and risk score. **(I–L)** The m6Ascore of ips_ctla4_neg_pd1_neg, ips_ctla4_neg_pd1_pos, ips_ctla4_pos_pd1_neg and ips_ctla4_pos_pd1_pos in two subgroups. Adjusted p-values were shown as *p<0.05; **p<0.01; ***p<0.001.

To further investigate the TME status in different subgroups, we utilized GSEA and found that the high-risk group was enriched in some cancer-related pathway and metastasis-related pathway, while the low-risk group was enriched in the pathways related to the immune disease (**Figures 6A, B** **)**. Following that, ssGSEA revealed that the low-risk group has high immune infiltration levels (**Figure 6C**). To further investigate the characteristic of these subtypes, we divided 220 TCGA patients into various immune subgroups. C2 is the most prevalent subtype and has the lowest risk score, while C1 has the highest risk score (**Figures 6D, E** **)**. It was observed that the risk score has negative correlation with estimated scores, immune scores, and stromal scores (**Figures 6F–H**). After comparing the TME scores of these two groups, we found that the low-risk group has higher estimated scores but lower immune score (**Figure 6I**). Subsequently, we further investigate the correlation between DRGs and immune cell abundance. A significant difference in the abundance of innate and adaptive immune cells was observed between the two risk groups (**Figures 7A–C**). Furthermore, just as **Figure 7D** illustrates, the ARG score was positively correlated with T cells CD4 memory resting, B cells naive, macrophages M2, mast cells activated, and neutrophils, but the opposite relationship was observed with T cells CD4 memory activated, T cells CD8, T cells gamma delta, T cells follicular helper, macrophages M1 and plasma cells. Then we explored the relationship between the selected ARGs in the prognostic signature and immune cells abundance, where the results indicate that many immune cells like T cells regulatory, T cells gamma delta, and T cells CD4 memory resting were strongly correlated with these genes, especially for the gene CXCL13 and GFRA1 (**Figure 7E**). Further research indicates that the low-risk group has higher HLA related genes expression levels and higher immune checkpoint expression levels (**Figures 7F, G** **)**. The six genes selected like CTLA4, HAVCR2, and CD274 were all negatively correlated with the risk score (**Figure 7H**). Moreover, IPS scores of patients were higher in low-risk groups, which indicates that they have higher immunogenicity (**Figures 7I–L**). Based on the above findings, it can be inferred that the low-risk group can be characterized as the “hot” tumor mentioned before.

### Relationships between ARG score and TMB

Numerous studies have demonstrated that tumor mutation burden (TMB) can be used to predict tumor immune response, thereby identifying patients who may benefit from ICIs (43, 44). Our results revealed that there were no difference between risk and TMB (**Figures S8A, B** **)**. Subsequently, to further investigate the impact of TMB on OV patients, we analyzed the survival probability in different TMB subgroups. The patients in the L-TMB group present a low survival probability compared with the H-TMB group (**Figure S8C**). Furthermore, we integrated the TMB and risk score for survival probability analysis, where the group with high TMB and low risk has the highest survival probability, while the group with low TMB and high risk was the lowest (**Figure S8D**). Next, we assessed the distribution of somatic mutations between two risk score subgroups in the TCGA-OV cohort. The mutation incidence of these two subgroups presented some similarity, where genes like TP53, TTN, MUC16, and CSMD6 all presented high alternations, especially for TP53 and TTN. However, except for the gene TTN, all these major mutated genes showed a higher alternation in the low-risk group (**Figures S8E, F**)

### Analysis of drug sensitivity

To further examine the efficacy of the ARG score as a marker predicting the therapeutic response of the patients, we calculated the sensitivity of different subgroups of patients to five chemotherapeutic agents commonly used in OV. As depicted in **Figure S9A**, IC50 values of three chemotherapeutic drugs chosen (gemcitabine, paclitaxel, and vinblastine) were lower in patients of the low-risk group, while the other two (bleomycin and docetaxel) were lower in patients of the high-risk group. Following that, we calculated the correlation between ARGs and different drugs, and the results further verified the associations between the ARGs and drug sensitivity (**Figure S9B**).

### Develop nomograms for survival predicting

**Figure 8:**
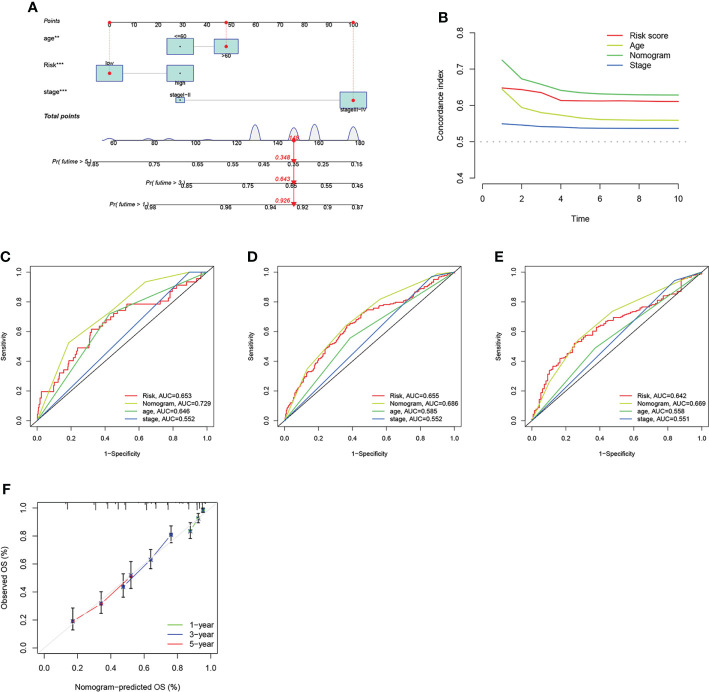
*Construction and validation of the nomogram.* **(A)** Nomogram constructed for predicting the clinical outcomes at 1-, 3, and 5- years for OV patients. **(B)** The consistency index of prognosis factors. **(C–E)** The ROC curves of the nomograms for 1-, 3-, 5- years OS in OV patients. **(F)** Nomogram calibration curve of 1 -, 3 -, and 5 years.

Given the important role of the risk score in the prognostic model, we incorporated it with clinical characteristics like age and stage to construct a nomogram, aiming to estimate the clinical outcomes for 1-, 3- and 5- years (**Figure 8A**). The C-index of the nomogram developed was higher than other models that only consider one clinical feature (**Figure 8B**). Subsequently, we estimated the AUC values of these models for predicting the clinical outcomes at 1-, 3- and 5- years, where the nomogram has the highest AUC values as we expected, indicating that the nomogram combined ARG risk score, age and stage has a better prediction performance (**Figures 8C–E**). The subsequent calibration diagram further validated the accurate prediction performance by comparing it with the actual OS observed (**Figure 8F**).

### ARG model as a new predictor of OV

To further demonstrate the predictive capacity of our model, we examined and compared three previously established OV prognostic models with our own (51–53). To make them comparable, multivariate analysis was employed to calculate the risk value of each dataset with these three published models. The analysis of survival probability revealed that the prognosis of the low-risk patients was much better in all of these three models (**Figures S10A–C**). However, the ROC curves indicate that the AUC value of our model was higher than these three models (**Figures S10D–F**). Then the C index was calculated utilizing the restricted mean survival (RMS) package, where we observed that the C index of our model was 0.621, higher than previously established models (**Figure S10G**). The above results make us convinced that our model has better prediction performance.

## Discussion

OV is a prevalent gynecological malignancy worldwide with the highest mortality (54). Every year, more than 240,000 women are diagnosed with OV, responsible for 150,000 deaths (55). The vast majority of ovarian cancer fatalities are attributable to the chemoresistant and widely metastatic disease in the late stage (56). Worse more, while the majority of patients will respond to first-line chemotherapy, disease recurrence rates remain high and the 5-year survival rate is extremely low (57–59). There is an urgent need for the development of novel therapeutic methods that take advantage of the diverse genetics and unique tumor microenvironment of a patient**’**s cancer. Angiogenesis is a natural and complicated process controlled by various biomolecules produced in the body. Effective suppression of tumor angiogenesis can help halt tumor progression, especially when combined with chemotherapy (60). Moreover, Numerous studies have demonstrated the inextricable link between intrinsic immunity and angiogenesis, and angiogenesis inhibiting may play a crucial role in boosting tumor immunotherapy (61, 62).

Therefore, it is of great significance to explore the role of angiogenesis in tumors, and many studies have established prognostic models to assess prognosis and immune microenvironment in OV (63, 64). The results of our study demonstrated the function of ARGs in (OV), where we screened two distinct molecular subgroups based on 48 ARGs and found that patients in ARGcluster B had superior survival and clinicopathological features. Subsequently, we investigate the feature of TME among these two subgroups, where ARGcluster A has a higher infiltration level of immune cells and is predicted to benefit more from immunotherapy. In addition, ARGs are predominately enriched in immune-related pathways, illustrating their substantial effect on the immunological regulation of TME. Subsequently, two gene clusters were identified based on DEGs, where the results demonstrated the potential of ARGs serving as predictors for the clinical outcomes and immunotherapeutic response of patients. Interestingly, patients in gene cluster A have higher immune infiltration levels, TME scores, and ARG expression levels but worse survival status. Gene cluster A can be identified as a **“**hot**”** tumor based on these markers, which corresponds to prior results of ARGclusters, as gene cluster A is an identical subset of the ARGcluster A.

Based on DEGs, we constructed an ARG-based prognostic model for individual OV patients. This model consists of TENM3, GFRA1, HOXA3, and CXCL13. A gene-based query at the Human Protein Atlas revealed the correlation between poor survival and high TENM3 expression in the majority of the examined malignancies, including ovarian, endometrial, and glioma cancer (65). In addition, immunotherapy has been developed in response to the identification of TENM3 as one of the neoantigens expressed in recurrent OV patients (66). GFRA1 plays a crucial role in the formation and maintenance of the nervous system, whose abnormal expression level is frequently observed in numerous cancer cells (67). Mounting evidence revealed the involvement of GFRA1 in the development and progression of tumors (68–70). HOXA3 is a member of the HOX transcription factor family, which regulates gene expression in embryonic development and performs crucial physiological functions. The expression of HOXA3 is associated with the immune system and cancer development, where it has been used as the diagnostic biomarker in various cancer (71–74). As for CXCL13, it has functions in inflammatory, infectious, and immune responses. CXCL13 is involved in the control of cancer cell phenotypes and play an important role in the progression and metastasis of solid tumor (75). Furthermore, a recent study revealed its function in maintaining the antitumor environment and supported clinical investigation on the combination of CXCL13 and PD-1 blockade therapy for high-grade serous ovarian cancer (76). Accordingly, the ARG score model comprised of these four genes has the potential to predict the clinical outcomes and immunotherapy response of OV patients.

TME refers to the ecosystem around the tumor in the body, which has been considered the crucial determinant in the incident and progression of OV (77, 78). Moreover, previous studies have highlighted TME reactive therapy as a promising strategy for developing accurate cancer-targeted therapies (79, 80). Therefore, we further investigate the TME status in two ARG score subgroups, where the results revealed that patients in the low-risk score group have a high estimated score, immune cells, and stromal cells. Moreover, the ARG score was negatively correlated with the abundance of some innate and adaptive immune cells like CD8+ T cells, T cells follicular helper, Gamma Delta T cells, and macrophages M1. CD8+ T cells often serve as the backbone of cancer immunotherapy for their prominence as anticancer immune response effectors (81, 82). The presence of T follicular helper cells in solid tumor tissue is indicative of a favorable prognosis, which is indispensable for the potent antibody responses of B cells (83). As the bridge between innate and adaptive immune systems, Gamma Delta T cells are involved in various immune responses during the progression of the tumor. Moreover, Gamma Delta T cells have received extensive attention in cancer immunotherapy for their antitumor cytotoxicity and potent cytokine production (84). Macrophage M1 has a pro-inflammatory effect, whose expression is positively correlated to the prognosis of patients with OV (85). Furthermore, some immune-related processes like CD8 T effector, antigen processing machinery, and Pan−F−TBRS were more prevalent in ARGcluster A and gene cluster A. Subsequently, we further investigate the discrepancies in the characteristics of TME and the abundance of 22 TIIC between subgroups, which illustrates the significance of ARGs in the OV progression.

Currently, the only treatment strategy for OV is cytoreductive surgery and platinum**/**taxane combined chemotherapy. Fortunately, immunotherapy has made great progress in gynecological malignancies, especially for ICIs (86). Moreover, a recent study demonstrated that combining immunotherapy with chemotherapy can considerably enhance treatment efficacy  (87). Further research revealed that the low-risk score group has higher HLA and immune checkpoint expression levels. Besides, the high IPS scores in the low-risk score group indicated higher immunogenicity. As for the six genes selected, CTLA4, HAVCR2 and CD274 had a negative correlation to the risk score. All these three genes have been well studied and proved to be important immunotherapeutic targets (88–91). TMB is regarded as a significant immunotherapy predictor, where multiple tumor instances demonstrated that the TMB score is positively correlated to the immunotherapy outcome, corresponding to our findings (92). The above results demonstrated that the low ARG score group is more suitable for immunotherapy.

The mutation rate of the two ARG score subgroups presented some similarity, where genes like TP53 (>80%), and TTN (>20%) all presented high alternations. These two genes have been demonstrated to play important roles in tumor progression and immune infiltration of TME in previous research (93–95). Nowadays, chemotherapy resistance remains a major challenge in the treatment of ovarian cancer (96). This study further investigated potentially sensitive agents in patients of different ARG score groups, which may help alleviate drug resistance and improve clinical outcomes. It was observed that the IC50 values of three chemotherapeutic drugs (gemcitabine, paclitaxel, and vinblastine) were lower in the low ARG score group, while the other two (bleomycin and docetaxel) were lower in the high ARG score group. Moreover, significant differences in drug sensitivity were detected between the two risk groups, where specific people identified can be treated with drugs of higher sensitivity.

Finally, we incorporated the ARG scores and clinical features like age and stage into a nomogram to illustrate the function of these factors in OV prognosis and thereby improve the clinical application of the ARG score. In this study, three previously established models were selected and their prediction performance was compared (51–53). Nevertheless, current research has limitations. All conclusions are based on the processing and analysis of public database data, but there is a dearth of clinical data and experimental studies to verify the results. Future research into the clinical applicability of the model will necessitate the collection of additional OV cases and the execution of a substantial number of prospective clinical evaluations.

## Conclusion

Our comprehensive analysis of ARGs successfully demonstrated its value in the field of TME, prognosis, and clinical characteristics of OV patients. Our study also highlights the value of ARGs in the prognostic model and their potency as the biomarker of the immunotherapy response. Our findings validate the great clinical significance of ARGs and provide new guidance for further research on personalized therapy strategies for OV patients.

## Data availability statement

The datasets presented in this study can be found in online repositories. The names of the repository/repositories and accession number(s) can be found in the article/**Supplementary Material**.

## Author contributions

SH, XZ and JW conceived the study and participated in the study design, performance and manuscript writing. HT, JS and JL conducted the bioinformatics analysis. XW and FW revised the manuscript. All authors read and approved the final manuscript.

## Acknowledgments

We would like to extend our gratitude to the researchers and study patients for their contributions.

## Conflict of interest

The authors declare that the research was conducted in the absence of any commercial or financial relationships that could be construed as a potential conflict of interest.

## Publisher’s note

All claims expressed in this article are solely those of the authors and do not necessarily represent those of their affiliated organizations, or those of the publisher, the editors and the reviewers. Any product that may be evaluated in this article, or claim that may be made by its manufacturer, is not guaranteed or endorsed by the publisher.

## Supplementary material

Supplementary Figure 1 The flow chart of this study.

Supplementary Figure 2 Correlation between the OS variations and gene expression level.

Supplementary Figure 3 Consensus clustering analysis. **(A)** Uniform clustering cumulative distribution function (CDF) with the number of clusters k, ranging from 2 to 9. **(B)** The change of area under CDF curve with k ranging from 2 to 9. **(C)** Tracking plot of the cluster when k = 2.

Supplementary Figure 4 Consensus clustering analysis. **(A)** Uniform clustering cumulative distribution function (CDF) with the number of clusters k, ranging from 2 to 9. **(B)** The change of area under CDF curve with k ranging from 2 to 9. **(C)** The samples were classified into two clusters when k = 2. **(D)** Tracking plot of the cluster when k = 2.

Supplementary Figure 5 Identification of representative candidate prognostic genes. **(A, B)** The LASSO regression analysis and partial likelihood deviance on the prognostic genes.

Supplementary Figure 6 Validation of the prognostic model based on ARG score. The expression patterns of eight genes between the two groups in testing set_GSE9891 **(A)**, testing set_GSE9891+TCGA **(B)**. The median risk score to separate the patients in testing set_GSE9891 **(C)**, testing set_GSE9891+TCGA **(D)**. The fustat of patients in testing set_GSE9891 **(E)** and testing set_GSE9891+TCGA **(F)** were shown in a scatter plot. Kaplan–Meier analysis reveals the difference of the survival probability between the two groups in testing set_GSE9891 **(G)**, testing set_GSE9891+TCGA **(H)**. ROC curves were performed to predict the sensitivity and specificity of 1-, 3- and 5-year survival according to the CRG score in testing set_GSE9891 **(I)**, testing set_GSE9891+TCGA **(J)**.

Supplementary Figure 7 Clinical correlation analysis and stratified analysis of the model. **(A–D)** Comparison of risk scores of patients with different ages, fustat, grade and stage. **(E–J)** Survival analysis of OV patients with various clinical characteristics.

Supplementary Figure 8
*Analysis of TMB in ARG score groups.* **(A, B)** Relationships between ARG scores and TMB. **(C)** Analysis of survival probability in different TMB groups. **(D)** Analysis of survival probability combined TMB and ARG scores. **(E, F)** The distribution of somatic mutations in two ARG score groups.

Supplementary Figure 9 Analysis of drug sensitivity. **(A)** The difference of bleomycin, docetaxel, gemcitabine, paclitaxel and vinblastine IC50 between high and low risk groups of patients, respectively. **(B)** Correlation between common drugs and ARGs

Supplementary Figure 10
*Comparison of our risk model with three published models.* **(A–C)** Analysis of survival probability for three published models. **(D–F)** ROC curves of three published models. **(G)** Comparison of the C-index in four models.
