# Supplementary figures and images for: Molecular subtypes, clinical significance, and tumor immune landscape of angiogenesis-related genes in ovarian cancer
Source: Front Oncol. 2022 Aug 29;12:995929. doi: 10.3389/fonc.2022.995929 (PMC9464911; doi:10.3389/fonc.2022.995929)

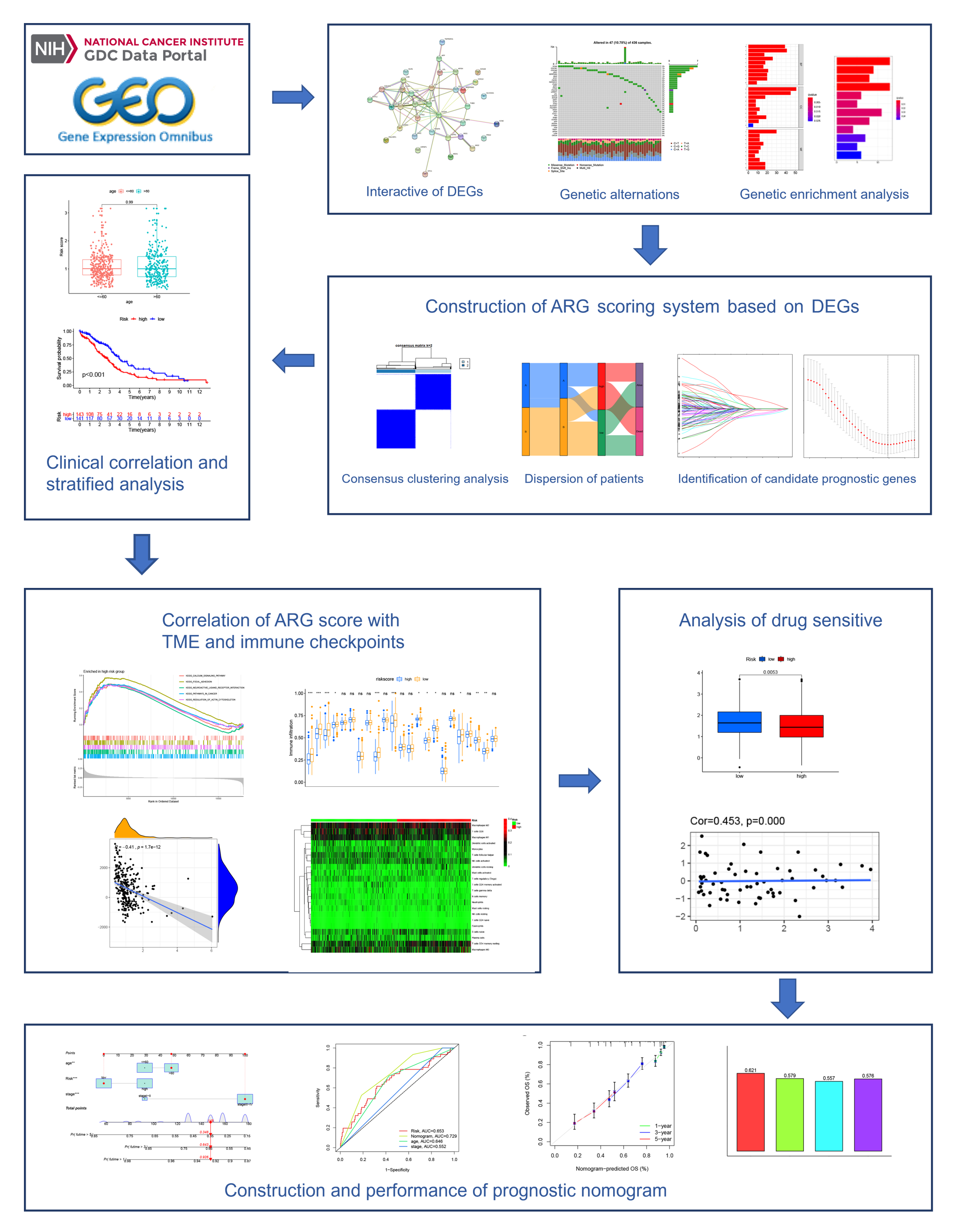

Supplement: Supplementary Figure 1 — The flow chart of this study. [file Image_1.tif]

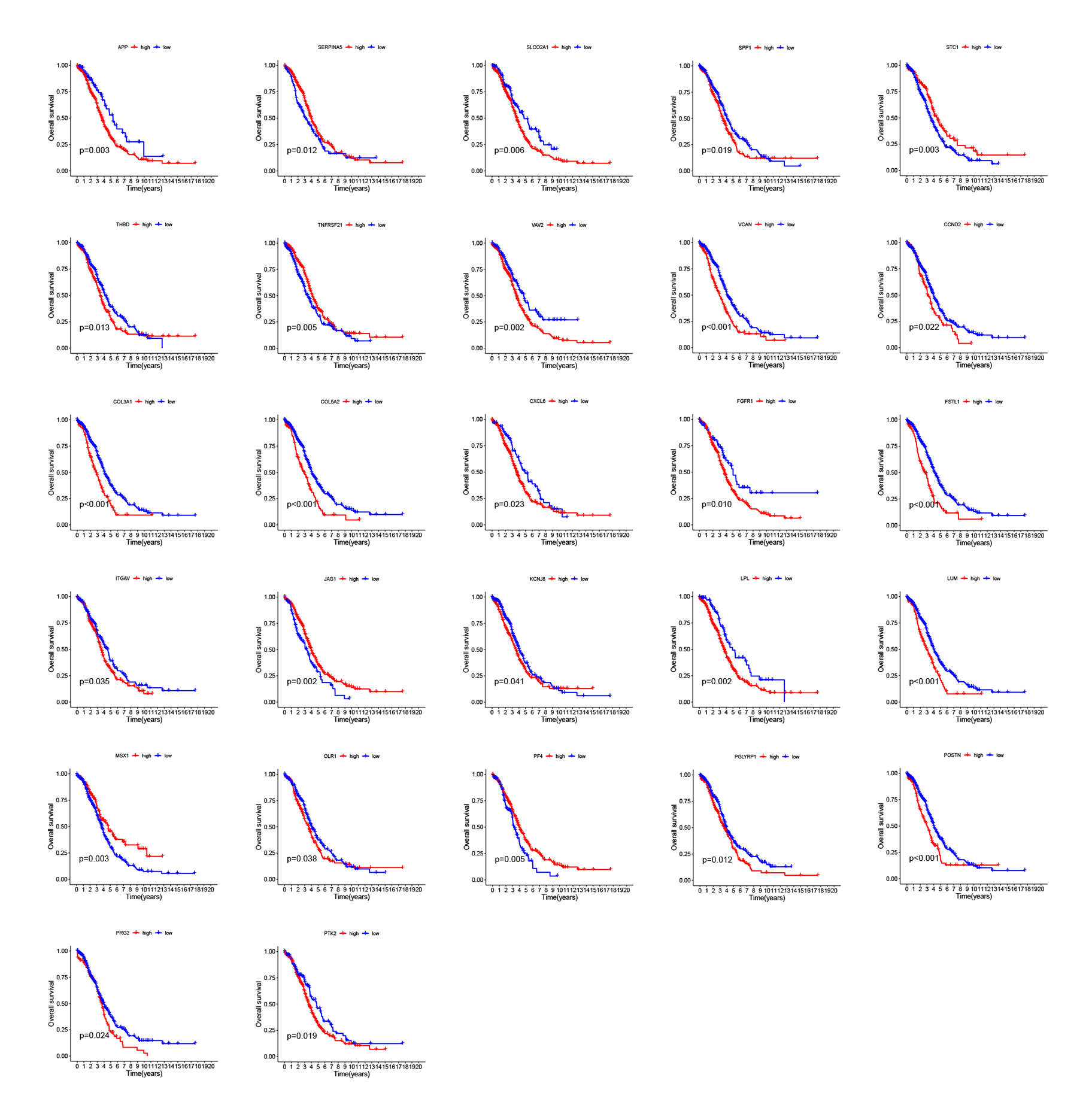

Supplement: Supplementary Figure 2 — Correlation between the OS variations and gene expression level. [file Image_2.tif]

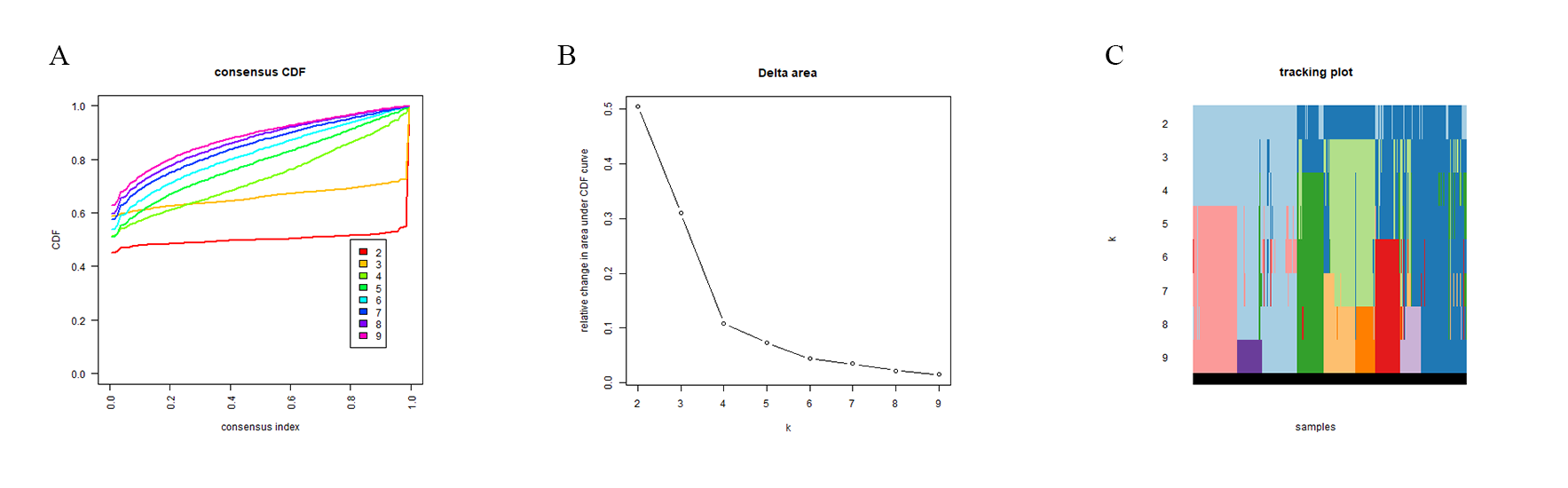

Supplement: Supplementary Figure 3 — Consensus clustering analysis. (A) Uniform clustering cumulative distribution function (CDF) with the number of clusters k, ranging from 2 to 9. (B) The change of area under CDF curve with k ranging from 2 to 9. (C) Tracking plot of the cluster when k = 2. [file Image_3.tif]

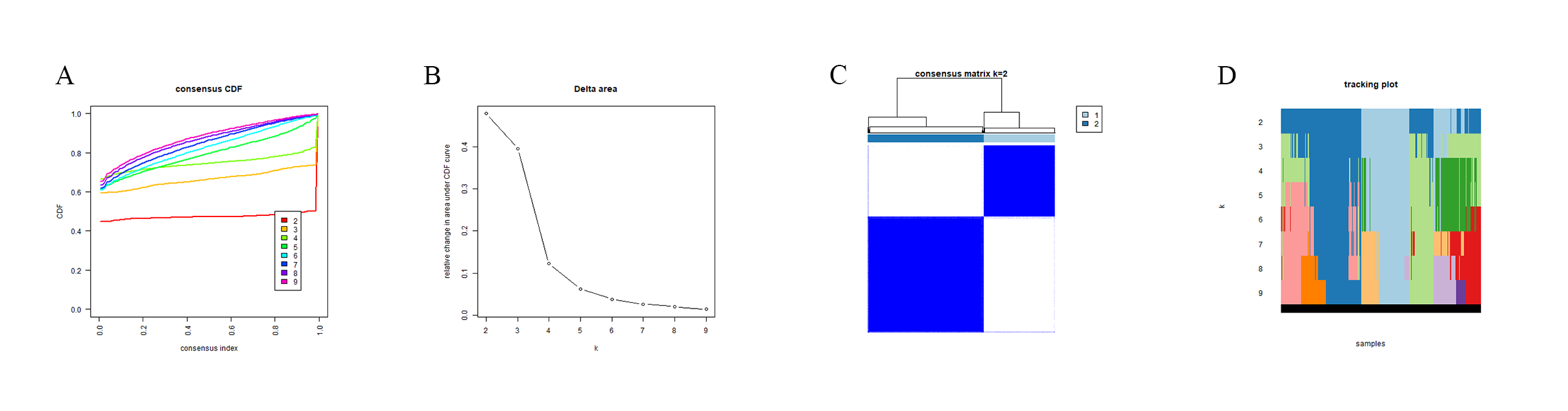

Supplement: Supplementary Figure 4 — Consensus clustering analysis. (A) Uniform clustering cumulative distribution function (CDF) with the number of clusters k, ranging from 2 to 9. (B) The change of area under CDF curve with k ranging from 2 to 9. (C) The samples were classified into two clusters when k = 2. (D) Tracking plot of the cluster when k = 2. [file Image_4.tif]

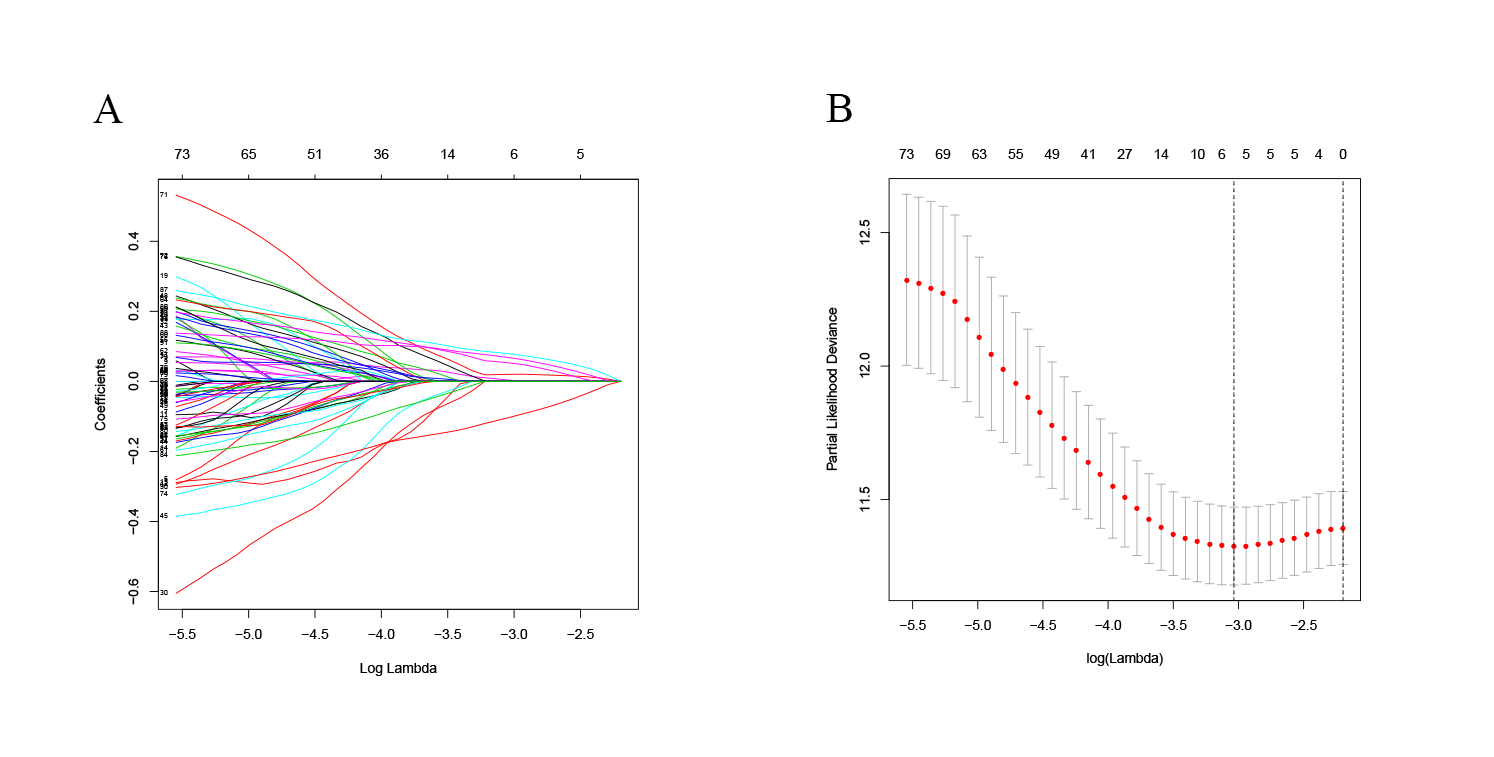

Supplement: Supplementary Figure 5 — Identification of representative candidate prognostic genes. (A, B) The LASSO regression analysis and partial likelihood deviance on the prognostic genes. [file Image_5.tif]

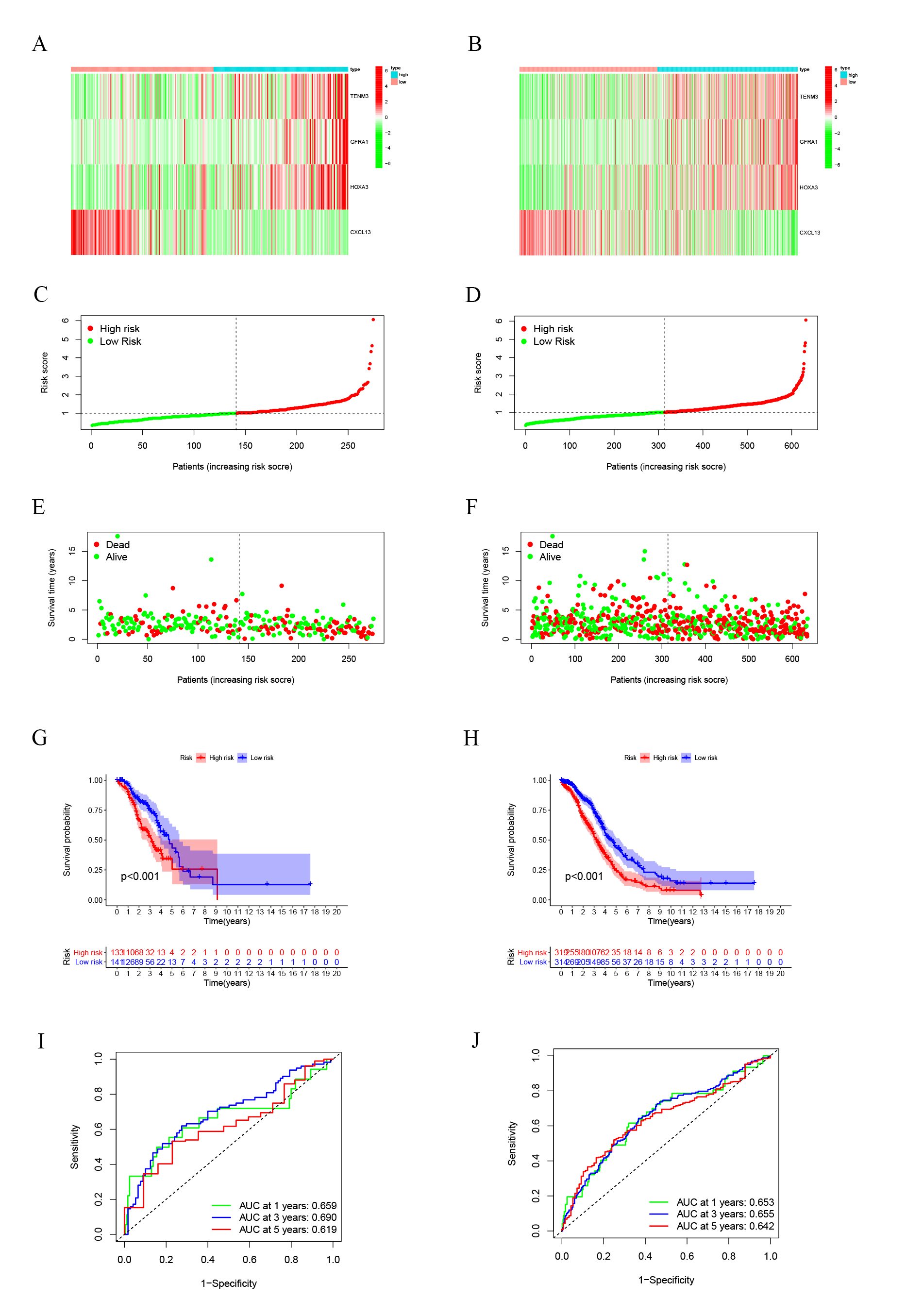

Supplement: Supplementary Figure 6 — Validation of the prognostic model based on ARG score. The expression patterns of eight genes between the two groups in testing set_GSE9891 (A), testing set_GSE9891+TCGA (B). The median risk score to separate the patients in testing set_GSE9891 (C), testing set_GSE9891+TCGA (D). The fustat of patients in testing set_GSE9891 (E) and testing set_GSE9891+TCGA (F) were shown in a scatter plot. Kaplan–Meier analysis reveals the difference of the survival probability between the two groups in testing set_GSE9891 (G), testing set_GSE9891+TCGA (H). ROC curves were performed to predict the sensitivity and specificity of 1-, 3- and 5-year survival according to the CRG score in testing set_GSE9891 (I), testing set_GSE9891+TCGA (J). [file Image_6.tif]

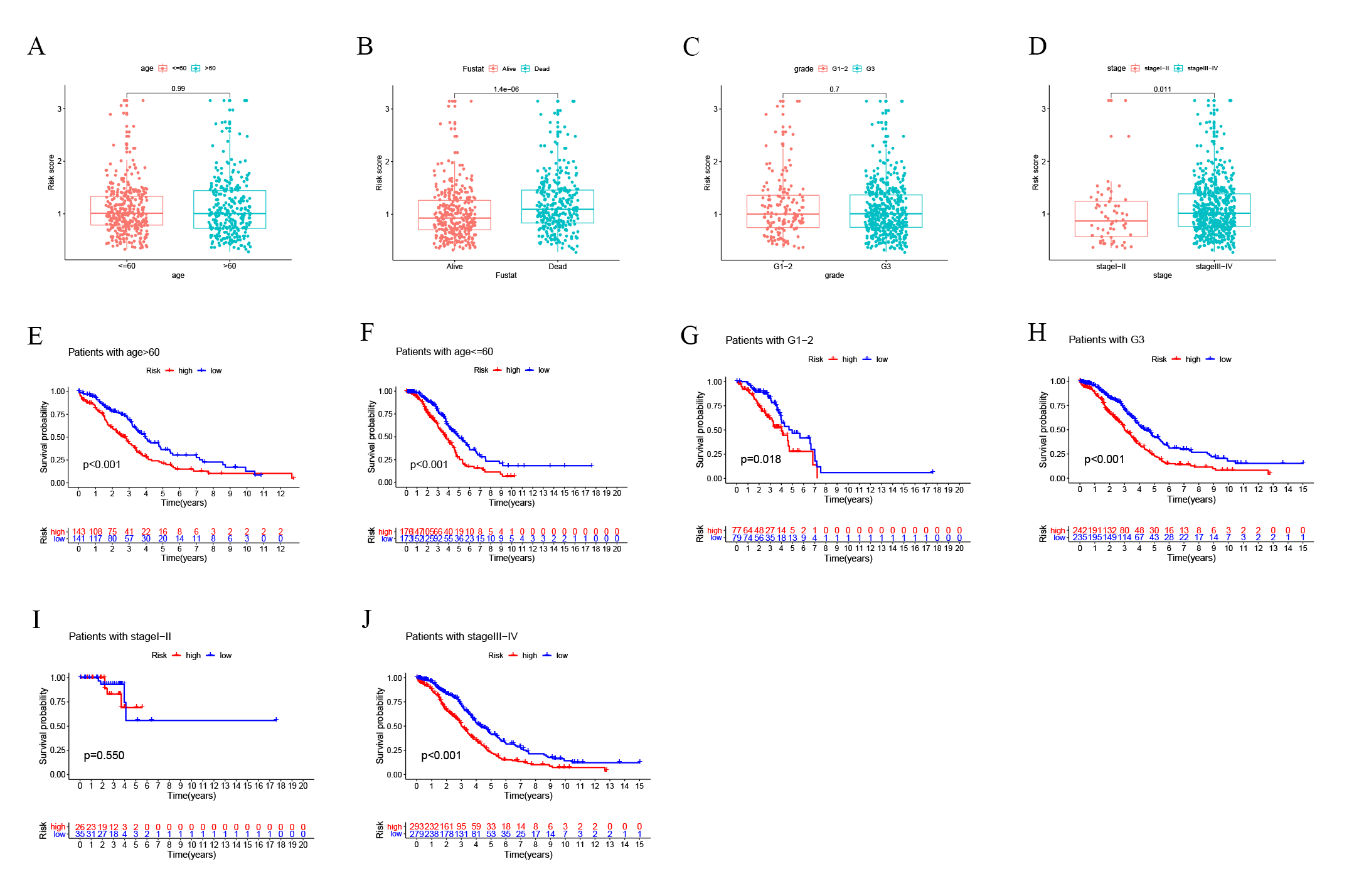

Supplement: Supplementary Figure 7 — Clinical correlation analysis and stratified analysis of the model. (A–D) Comparison of risk scores of patients with different ages, fustat, grade and stage. (E–J) Survival analysis of OV patients with various clinical characteristics. [file Image_7.tif]

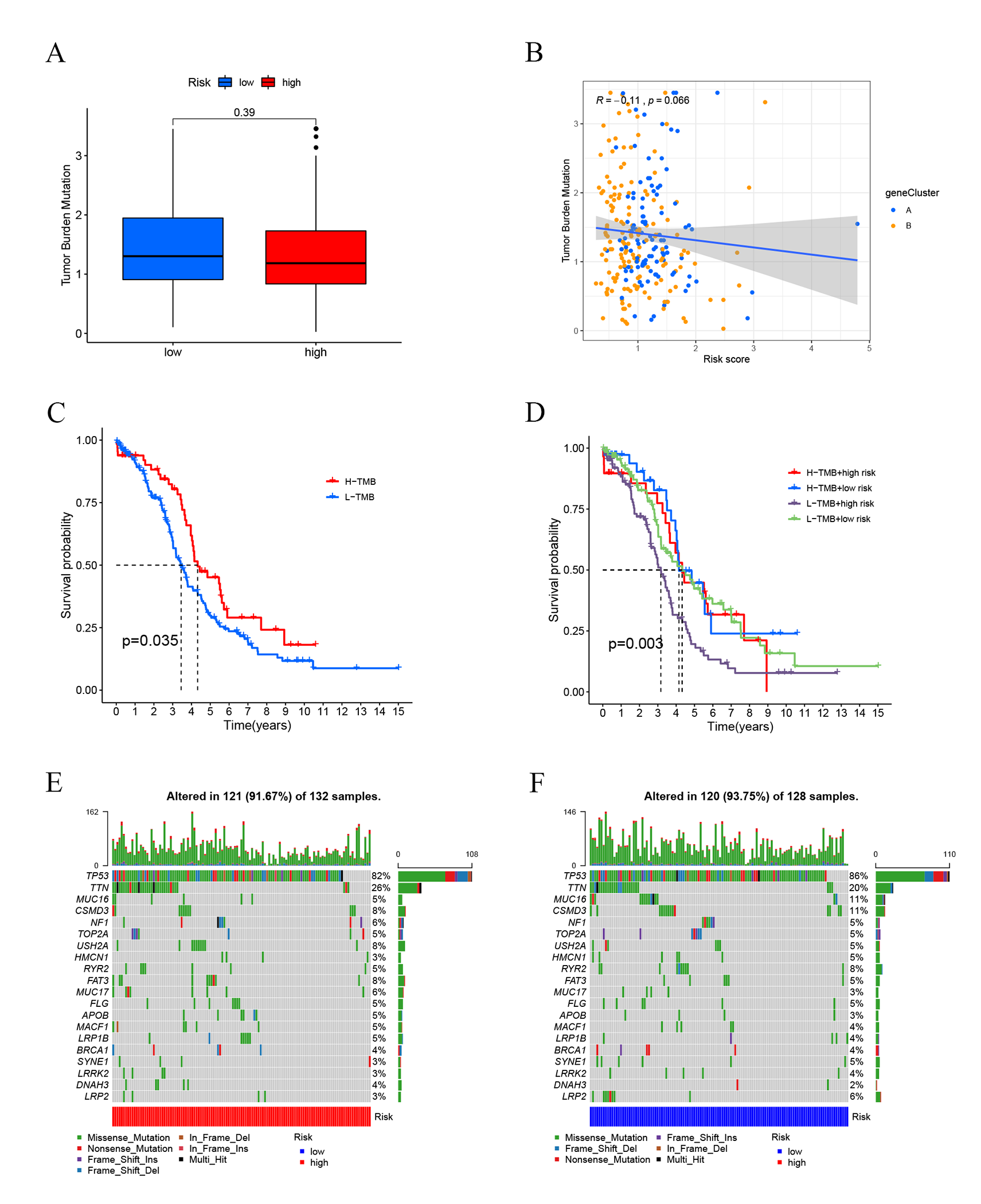

Supplement: Supplementary Figure 8 — Analysis of TMB in ARG score groups. (A, B) Relationships between ARG scores and TMB. (C) Analysis of survival probability in different TMB groups. (D) Analysis of survival probability combined TMB and ARG scores. (E, F) The distribution of somatic mutations in two ARG score groups. [file Image_8.tif]

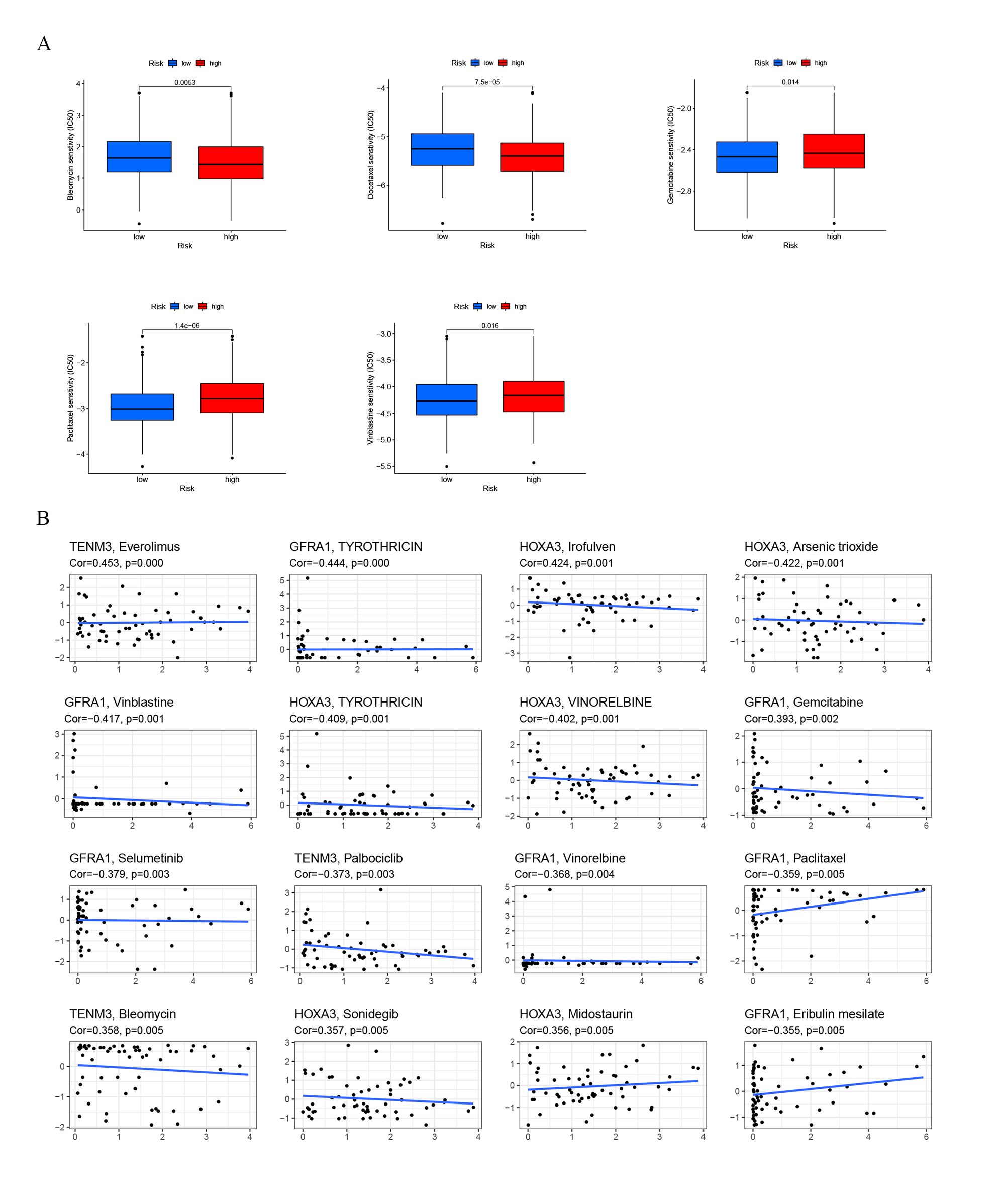

Supplement: Supplementary Figure 9 — Analysis of drug sensitivity. (A) The difference of bleomycin, docetaxel, gemcitabine, paclitaxel and vinblastine IC50 between high and low risk groups of patients, respectively. (B) Correlation between common drugs and ARGs [file Image_9.tif]

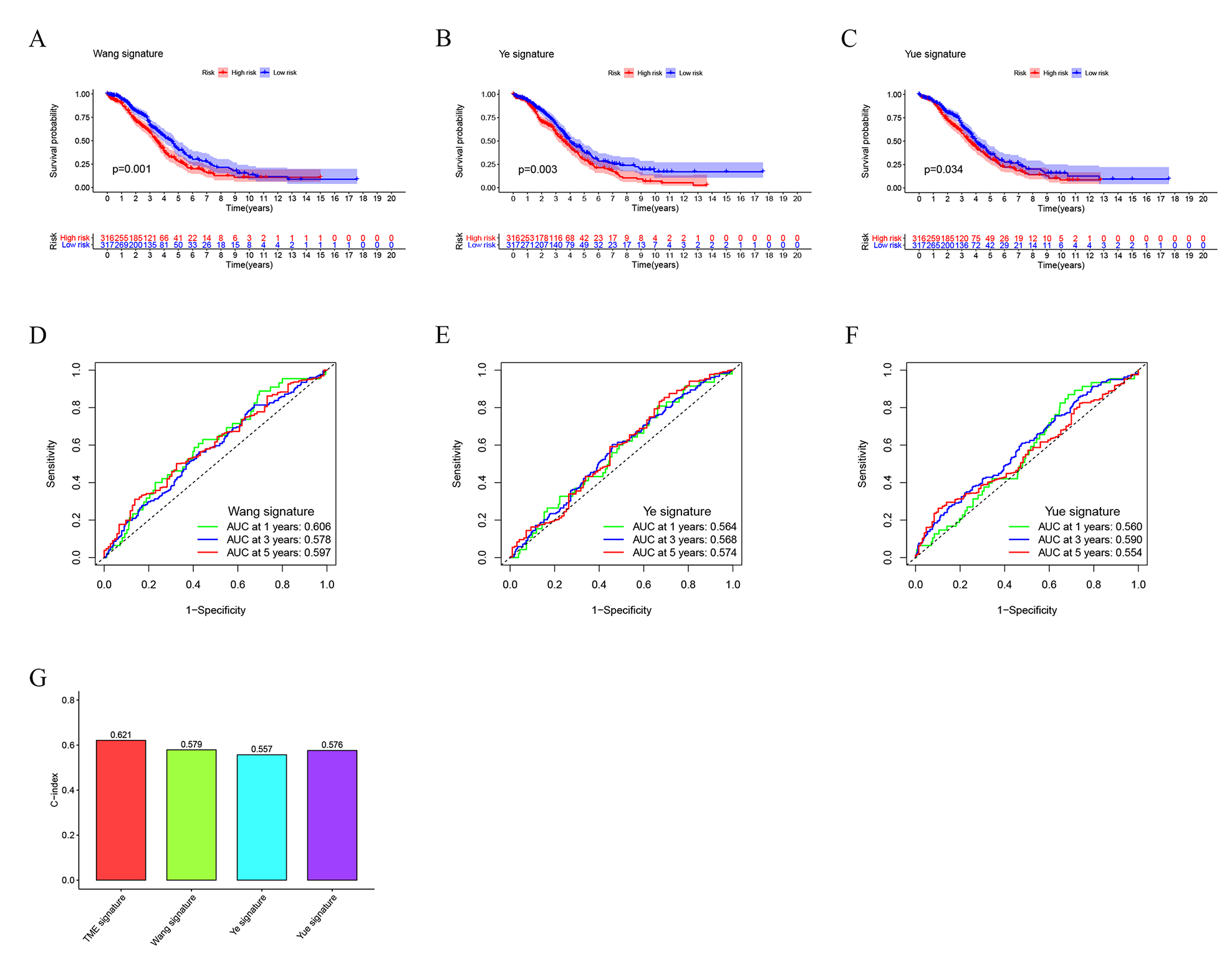

Supplement: Supplementary Figure 10 — Comparison of our risk model with three published models. (A–C) Analysis of survival probability for three published models. (D–F) ROC curves of three published models. (G) Comparison of the C-index in four models. [file Image_10.tif]
