# Supplementary material for: Molecular subtypes, clinical significance, and tumor immune landscape of angiogenesis-related genes in ovarian cancer
Source: Front Oncol. 2022 Aug 29;12:995929. doi: 10.3389/fonc.2022.995929 (PMC9464911; doi:10.3389/fonc.2022.995929)
Supplement: Supplementary file 11 [file Table_1.docx]

**Table S1** The clinical characteristics of OV patients in the ALL set.

| Covariates | Type | Total |
| --- | --- | --- |
| fustat | Alive | 432 (51.86%) |
| fustat | Dead | 401 (48.14%) |
| age | <=60 | 461 (55.34%) |
| age | >60 | 372 (44.66%) |
| grade | G1-2 | 188 (22.57%) |
| grade | G3 | 645 (77.43%) |
| stage | Stage I-II | 83 (9.96%) |
| stage | Stage III-IV | 750 (90.04%) |
