# Supplementary material for: Molecular subtypes, clinical significance, and tumor immune landscape of angiogenesis-related genes in ovarian cancer
Source: Front Oncol. 2022 Aug 29;12:995929. doi: 10.3389/fonc.2022.995929 (PMC9464911; doi:10.3389/fonc.2022.995929)
Supplement: Supplementary file 12 [file Table_2.docx]

| Variable | Univariable model | | | | Multivariable model | | | |
| --- | --- | --- | --- | --- | --- | --- | --- | --- |
|  | HR | HR.95L | HR.95H | pvalue | HR | HR.95L | HR.95H | pvalue |
| Training set |  |  |  |  |  |  |  |  |
| age | 1.3646 | 1.0140 | 1.8364 | 0.0402 | 1.2796 | 0.9483 | 1.7268 | 0.1068 |
| grade | 1.1042 | 0.7773 | 1.5687 | 0.5800 |  |  |  |  |
| stage | 3.5344 | 1.4507 | 8.6113 | 0.0055 | 3.1566 | 1.2949 | 7.6949 | 0.0115 |
| riskScore | 2.0009 | 1.5949 | 2.5102 | 0.0000 | 1.9414 | 1.5415 | 2.4451 | 0.0000 |
| Testing set |  |  |  |  |  |  |  |  |
| age | 1.5707 | 1.1468 | 2.1512 | 0.0049 | 1.4952 | 1.0881 | 2.0548 | 0.0131 |
| grade | 1.4607 | 0.9500 | 2.2459 | 0.0843 |  |  |  |  |
| stage | 4.9857 | 1.5866 | 15.6672 | 0.0060 | 6.2078 | 1.9198 | 20.0730 | 0.0023 |
| riskScore | 1.4142 | 1.1867 | 1.6854 | 0.0001 | 1.5696 | 1.2553 | 1.9625 | 0.0001 |

**Table S2** Cox regression analyses of the angiogenesis-related factors.
